# Association between Cyclothymic Affective Temperament and Age of Onset of Hypertension

**DOI:** 10.1155/2019/9248247

**Published:** 2019-11-18

**Authors:** Beáta Körösi, Milán Vecsey-Nagy, Márton Kolossváry, Zsófia Nemcsik-Bencze, Bálint Szilveszter, Andrea László, Dóra Batta, Xénia Gonda, Béla Merkely, Zoltán Rihmer, Pál Maurovich-Horvat, Dániel Eörsi, Péter Torzsa, János Nemcsik

**Affiliations:** ^1^Department of Family Medicine, Semmelweis University, Budapest, Hungary; ^2^MTA-SE Cardiovascular Imaging Research Group, Heart and Vascular Center of Semmelweis University, Budapest, Hungary; ^3^Magnetic Resonance Imaging Research Center, Semmelweis University, Budapest, Hungary; ^4^TCM-Klinik Bad Kötzting, Bad Kötzting, Germany; ^5^Department of Psychiatry and Psychotherapy, Semmelweis University, Budapest, Hungary; ^6^MTA-SE Neurochemistry Research Group, Budapest, Hungary; ^7^Heart and Vascular Center, Semmelweis University, Budapest, Hungary; ^8^Health Service of Zugló (ZESZ), Budapest, Hungary

## Abstract

Affective temperaments represent a biologically stable core of emotional reactivity and have previously been associated with hypertension and arterial stiffening. The age, when hypertension is initiated, is influenced by different factors, but the role of personality traits in this regard is not clarified yet. Our aim was to study the association between affective temperaments and the age at onset of hypertension. In this cross-sectional study, 353 patients were included. After the evaluation of history, patients completed the Temperament Evaluation of Memphis, Pisa, Paris and San Diego Autoquestionnaire. We used linear regression analysis to identify predictors of the age of onset of hypertension in the whole cohort and in male and female subpopulations. The independent predictors of the age at onset of hypertension were male sex (*B* = −4.57 (95% CI = −1.40 to −7.74)), smoking (*B* = −4.31 (−7.41 to −1.22)), and positive family history (*B* = −6.84 (−10.22 to −3.45)). In women, cyclothymic temperament score was an independent predictor of the initiation of hypertension (*B* = −0.83 (−1.54 to −0.12)), while this association was absent in men. Besides traditional factors, cyclothymic affective temperament might contribute to the earlier initiation of hypertension in women.

## 1. Introduction

Hypertension has an excessive impact on mortality worldwide. High blood pressure is the leading cause of death and disability-adjusted life years [1]. In the United States, hypertension accounted for more cardiovascular (CV) deaths than any other modifiable CV disease (CVD) risk factor and was second only to cigarette smoking as a preventable cause of death for any reason [2, 3].

There are modifiable (e.g., smoking, obesity, and unhealthy diet) and nonmodifiable (e.g., positive family history, age, and male sex) risk factors of CVD which have a complex and interdependent relationship with hypertension. CVD risk factors can influence those pathophysiological pathways which are also involved in the development of hypertension, like the renin-angiotensin-aldosterone system, sympathetic nervous system, cardiac natriuretic peptide system, or the endothelial function [4–6]. Consequently, hypertension can also be considered like an outcome of different harmful processes, not only a simple disease. As the development of hypertension in longer term can lead to adverse cardiovascular outcomes [7], the age at the onset of hypertension is important with respect to further management of patients.

Affective temperament types (depressive, cyclothymic, hyperthymic, irritable, and anxious) are subclinical, trait-related manifestations and commonly the antecedents of minor and major mood disorders [8]. Temperament is regarded as the inherited part of personality and represents a biologically stable core of emotional reactivity [9], although there is an ongoing discussion regarding the influence of age on depressive temperament with differences between men and women [10]. Affective temperaments can be measured on five temperament scales by the Temperament Evaluation of Memphis, Pisa, Paris and San Diego Autoquestionnaire (TEMPS-A) [11]. Hyperthymic temperament is characterized by upbeat, overconfident, and overenergetic traits, while depressive temperament is self-denying, striving to live in harmony with others, and sensitive to suffering. Anxious temperament can best be explained by exaggerated worries especially toward family members. Cyclothymic temperament shows affective instability with rapid mood shifts and intense emotions, while irritable temperament incorporates skeptical and critical traits [12–14].

Recently, association has been demonstrated between cyclothymic affective temperament score and brachial systolic blood pressure [15], and dominant cyclothymic temperament showed correlation with chronic hypertension and with acute coronary events in hypertensive patients [14, 16].

Our aim was to study the effect of affective temperaments on the time of onset of hypertension. As previously significant differences were found in the pattern of temperaments between men and women [10], we aimed to study the associations in the whole cohort and in the different genders separately as well. We hypothesized that high cyclothymic score represents a propensity for early development of hypertension.

## 2. Methods

### 2.1. Subjects

In a cross-sectional design, Caucasian hypertensive patients were involved into the study. Primary newly diagnosed hypertensive patients were involved from one primary care practice, but as their low number limited the detailed statistical analysis, patients with chronic hypertension were also included from the same primary care practice, from two others, and from a cardiovascular imaging outpatient clinic, all located in Budapest, Hungary. Patients were collected consecutively between January 2014 and May 2017. Previous papers were published on these cohorts [15, 17–19]. Inclusion criteria included patients older than 18 years, who gave approval to the study. In newly diagnosed hypertensive patients, hypertension was defined as office values >140 mmHg systolic blood pressure and/or >90 mmHg diastolic blood pressure, based on the actual European hypertension guideline [20]. In chronic hypertensive patients, the time of the onset of hypertension was based on the self-report of the patients, but it was confirmed by the medical records where it was possible. Patients with confirmed secondary hypertension, with ongoing psychiatric disorders or with dementia potentially interfering with the completion of questionnaires, were excluded from our study.

Patients were asked to complete an *autoquestionnaire* to evaluate the demographic, family, and medical history data and affective temperaments.

Newly diagnosed hypertensive patients were free of antihypertensive medication at the time of the completion of the psychometric questionnaires, as the questionnaires were given to the patients before the confirmation of the diagnosis of hypertension with ambulatory or home blood pressure monitoring and before antihypertensive medication initiation. A smaller proportion of chronic hypertensive patients had only lifestyle change recommendations, but most of them regularly took medications.

Prior to participation, all patients gave written informed consent. The study was approved by the Scientific and Research Ethics Committee of the Medical Research Council, the Hungarian Ministry of Health (ETT TUKEB 570/2014), and was carried out in accordance with the tenets of the Declaration of Helsinki.

### 2.2. Evaluation of Affective Temperaments

The *Temperament Evaluation of Memphis*, *Pisa*, *Paris and San Diego Autoquestionnaire* (TEMPS-A) was used to assess affective temperaments on depressive, cyclothymic, hyperthymic, irritable, and anxious subscales, requiring “yes” (score 1) or “no” (score 0) answers [11]. TEMPS-A contains 110 items (109 in the version for males), and the questions of the various temperament types are grouped together as follows:

1. Depressive temperament: questions 1 to 21 (21 points)
2. Cyclothymic temperament: questions 22 to 42 (21 points)
3. Hyperthymic temperament: questions 23 to 63 (21 points)
4. Irritable temperament: questions 64 to 84 (21 points in women and 20 in the men's version)
5. Anxious temperament: questions 85 to 110 (26 points)

TEMPS-A has been extensively studied, translated into more than 25 languages, and validated in several of the latter. Similarities and differences were also found in national samples, which suggest that distribution of affective temperaments has both universal- and cultural-specific characteristics [10].

### 2.3. Statistical Analysis

Descriptive data are expressed as mean ± standard deviation or percentages. Normality of continuous parameters was tested with the Kolmogorov–Smirnov test. Differences in variables between male and female patients were analyzed using unpaired Student's *t*-tests.

Linear regression analysis was used to study the determinants of the age at onset of hypertension first in the whole cohort. Variables considered in the model were age, sex, positive family history, smoking at the beginning of hypertension, alcohol consumption at the beginning of hypertension, and the five affective temperaments. Next, linear regression analysis was performed separately in men and women with all other variables considered in case of the whole cohort. A two-sided *p* < 0.05 was considered to be significant. SPSS (Armonk, NY, USA version 24.0) was used for all calculations.

## 3. Results

A total of 353 patients were involved into the study with newly diagnosed (*n* = 55) or chronic (*n* = 298) hypertension (186 female, 52.7%). 234 patients were involved form the three primary care practices and 119 patients from the cardiovascular imaging outpatient clinic. In chronic hypertensive patients, the exact time of the development of hypertension could have been confirmed by medical records only in 38 more cases, as in most of the time only the initiation of antihypertensive medication could have been identified which is often not equal with the time of the initiation of hypertension.  Table 1 summarizes patient characteristics and the scores of the different directions of the affective temperaments in the whole population and in men and women separately. In case of male patients, hypertension started earlier, and the ratio of men with alcohol consumption at the time of hypertension initiation was higher compared with women. In women, depressive and anxious temperament scores were higher and hyperthymic and irritable scores were lower compared with men. There has been a tendency of higher cyclothymic score in women, but it did reach only borderline significance.

The 55 newly diagnosed hypertensive patients were free of antihypertensive medication at the time of the completion of the psychometric questionnaires. Among chronic hypertensive patients, only lifestyle changes were recommended for 22 patients (7.3%). Among those chronic hypertensive patients who used antihypertensive medication, 151 patients (54.3%) took regularly ACE inhibitors, 89 patients angiotensin II receptor blockers (32%), and 122 patients calcium-channel blockers (43.9%). 118 patients (42.4%) were on diuretic, 180 patients (64.7%) on beta-blocker, 29 patients (10.4%) on alpha-blocker, 4 patients (1.4%) on centrally acting agent (rilmenidine), and 3 patients (1%) on nitrate donor (dihydralazine) therapy. 48 patients (17.2%) of the chronic hypertensive patients were on monotherapy, 106 of them (38.1%) took two, 73 of them (26.2%) three, 29 of them (10.4%) four, 19 of them (6.8%) five, 2 of them (0.7%) six, and one of them (0.3%) seven medications of different antihypertensive drug classes.

With multiple linear regression analysis, in the whole cohort, the independent predictors of earlier onset of hypertension were male sex (*B* = −4.57 (95% CI: −1.40–7.74), *p*=0.005), positive family history (*B* = −6.84 (95% CI: −10.22–3.45), *p* > 0.001), and smoking (*B* = −4.31 (95% CI: −7.41–1.22), *p*=0.006). Cyclothymic affective temperament had a tendency to be an independent predictor, but the association was not significant (*B* = −0.41 (95% CI: −0.99–0.17) *p*=0.163). Depressive (*B* = 0.29 (95% CI: −0.31–0.88) *p*=0.343), hyperthymic (*B* = 0.02 (95% CI: −0.34–0.37) *p*=0.928), irritable (*B* = 0.20 (95% CI: −0.28–0.53) *p*=0.530), and anxious temperaments (*B* = 0.13 (95% CI: −0.28–0.3) *p*=0.532) had no association with the age at the onset of hypertension.  Table 2 summarizes the results of the multiple linear regression analyses in the separated sexes. In men, positive family history and smoking remained independent predictors of the time of hypertension initiation; however, no association was found with any affective temperaments. However, in case of women, besides positive family history, cyclothymic affective temperament was found to be independently and inversely associated with the time of hypertension initiation. One point higher cyclothymic score was associated with 0.8 years earlier appearance of hypertension.

## 4. Discussion

Our study demonstrated that there is an inverse association between the time of onset of hypertension and cyclothymic affective temperament score in women, while this association is absent in men.

Affective temperaments, especially cyclothymic temperaments, have complex associations with psychopathology. Cyclothymic is the most pronounced affective temperament type in patients with bipolar II disorder [21, 22], and it is the precursor of bipolar disorder often with earlier onset, atypical features, more relapses, and worse prognosis [8, 23]. Interestingly, in a smaller part of “unipolar” depressive patients—especially with a positive family history of bipolar disorder—the core feature of cyclothymic temperament can also be detected with its typical mood and energy alterations [24]. In addition, cyclothymic temperament was also shown to be associated with atypical depression [25]. On the contrary, besides its well-described role in psychopathology, cyclothymic temperament seems to have importance also in relation of cardiovascular pathology, especially hypertension [14, 15, 17].

More pronounced cyclothymic affective temperament probably does not directly lead to the earlier development of hypertension, but there are different levels of crossroads that help in the deterioration of vascular physiology. First, it can be an association of cyclothymic affective temperament with modifiable risk factors of hypertension. Previously, it has been demonstrated that patients with marked cyclothymic and irritable temperaments show higher propensity for smoking [26]. In relation of morbid obesity dominant cyclothymic, irritable and anxious temperaments were found with higher prevalence compared with healthy controls [27]. In case of alcohol dependency, alcoholics scored higher on cyclothymic, depressive, and irritable scales [28]. Furthermore, cyclothymic and irritable scores were found to be higher in alcoholics in another recent study as well [29]. In our study, we adjusted our results for smoking and alcohol consumption, and cyclothymic temperament score remained a predictor of the time of initiation of hypertension. This finding proposes the mediator role of other factors as well.

Besides the association with risk factors of hypertension, direct connections of affective temperaments with pathophysiological pathways can also explain our findings. The neuroprotective serum brain-derived neurotrophic factor (BDNF) level was found to be lower in chronic hypertensive patients with dominant cyclothymic, irritable, depressive, or anxious temperaments compared with chronic hypertensive patients without dominant temperaments [17]. There is a close association between cyclothymic and irritable affective temperaments [30], and the latter shows similarities with anger and hostility traits. Highly hostile subjects have been found to exhibit increased catecholamine and cortisol secretion in response to anger-provoking stimuli [31], and they secrete increased levels of cortisol during daily living as well [32]. Hostility in healthy young adults has been reported as being inversely related to the high frequency components of heart rate variability power spectrum [33], which is under the regulation of the parasympathetic system [34, 35]. These results suggest the overactivation of the sympathetic nervous system in hostility which can lead to the development of hypertension. As there is a clear association between cyclothymic and irritable affective temperaments and consequently probably also between cyclothymic temperament and hostility, these mechanisms theoretically can also contribute our observed association between cyclothymic temperament and the initiation of hypertension, but these hypotheses need further studies to be confirmed.

In our study, the association between the initiation of hypertension and cyclothymic affective temperament has been demonstrated only in women. Sex differences in relation of the affective temperaments are already known from the literature. In a study of Vázquez et al., higher hyperthymic and irritable scores were described in men, while women scored superior in anxious, depressive, and cyclothymic directions [10]. In our study, we were almost able to totally reproduce these findings, although in case of cyclothymic temperament score there was only a tendency of difference between men and women, but it did not reach the level of significance.

Sex differences have also already been described in relation to the association between personality traits and cardiovascular pathology. Trait anger was found to be associated with carotid arterial stiffening only in men [35], and interaction has also been found between sex and different affective temperaments in the prediction of brachial systolic blood pressure or pulse wave velocity in chronic hypertensive patients [15]. The clarification of the pathophysiological background of this phenomenon needs further studies which also consider the use of oral contraceptives or menopausal status.

A main limitation of our study was that only 55 patients of the whole study population were involved at the time of the diagnosis of hypertension. In 38 more chronic hypertensive cases, it could have been evaluated exactly, but in the rest 280 patients, we had to rely on the self-report about the time of onset of hypertension. From the same reason, anthropometric data (like body weight), laboratory results, and the level of depression or anxiety were not available in most of the patients from the time of the initiation of hypertension; therefore, these factors were not involved into the multiple regression analyses. So later this study should be reproduced on patients involved at the time of the initiation of hypertension and considering the abovementioned factors. However, as risk factors which are proven to play a role in the initiation of hypertension, like male sex, smoking, and positive family history, were reproduced in our study, this gives an internal validation to our results and suggests that the self-report of the patients was acceptably exact. Another limitation is that, in most of the patients, affective temperaments were evaluated years after the initiation of hypertension. However, as temperaments are relatively stable in adulthood [36], we think our results can give a good estimation about the association of affective temperaments with the initiation of hypertension. In addition, our subjects were composed of subjects with first evaluation and subjects with previous diagnosis of hypertension with or without ongoing antihypertensive medication; however, we think that based on the stability of affective temperaments, this fact did not influence markedly the final outcome. Moreover, although our methodology used standardized autoquestionnaires and excluded patients with dementia, a complete exclusion of misinterpretations or mistakes by patients is nevertheless impossible. Finally, a limitation stems from the cross-sectional design of the study which precludes causal inference.

In conclusions, our results demonstrated that cyclothymic affective temperament score has an inverse association with the initiation of hypertension in women and thus could have an influence for cardiovascular pathology, and with more supporting data in the future, it might be considered as an independent risk factor of hypertension. It seems that the evaluation of affective temperaments could have an importance not only in psychopathology but also in cardiovascular prevention.

## Figures and Tables

**Table 1:** Patient characteristics and the scores of the different directions of the affective temperaments in the whole population and in men and women separately.

	Total	Male patients	Female patients	*p*
	*N* = 353	*N* = 167	*N* = 186	
Age (years)	**60.8** **±** **12.0**	**57.9** **±** **13.1**	**63.3** **±** **10.4**	**<0.001**
Initiation of hypertension (years)	**48.5** **±** **13.5**	**46.3** **±** **13.8**	**50.4** **±** **12.9**	**0.004**
Positive family history, *n* (%)	269 (76.2)	121 (72.5)	148 (79.6)	0.092
Smoking at hypertension initiation, *n* (%)	118 (33.4)	54 (32.1)	64 (34.4)	0.680
Alcohol consumption at hypertension initiation, *n* (%)	**162 (45.9)**	**99 (59.3)**	**63 (33.9)**	**<0.001**

*TEMPS-A*				
Depressive	**6.7** **±** **3.3**	**5.9** **±** **2.8**	**7.5** **±** **3.5**	**<0.001**
Cyclothymic	4.1 ± 4.0	3.8 ± 3.8	4.5 ± 4.1	0.092
Hyperthymic	**11.5** **±** **4.3**	**12.3** **±** **4.3**	**10.8** **±** **4.1**	**0.001**
Irritable	**4.4** **±** **3.3**	**5.0** **±** **3.6**	**3.9** **±** **2.9**	**0.001**
Anxious	**6.2** **±** **5.7**	**4.7** **±** **5.4**	**7.6** **±** **5.7**	**<0.001**

Continuous data are presented as mean ± SD. TEMPS-A, Temperament Evaluation of Memphis, Pisa, Paris and San Diego Autoquestionnaire.

**Table 2:** Results of the multiple linear regression analyses in the separated sexes.

****	*B*	Std error	*p*	Lower bound	Upper bound
Hypertensive men positive family history	**−6.73**	**2.41**	**0.006**	**−11.49**	**−1.97**
Alcohol	−2.21	2.25	0.327	−6.64	2.23
Smoking	**−6.97**	**2.33**	**0.003**	**−11.57**	**−2.36**

*TEMPS-A*					
Depressive	−0.38	0.47	0.424	−1.31	0.55
Cyclothymic	0.26	0.49	0.595	−0.71	1.23
Hyperthymic	−0.31	0.26	0.225	−0.82	0.19
Irritable	0.44	0.46	0.342	−0.47	1.34
Anxious	−0.32	0.31	0.310	−0.93	0.30

*Hypertensive women*					
Positive family history	**−5.87**	**2.48**	**0.019**	**−10.77**	**−0.97**
Alcohol	3.29	2.13	0.125	−0.92	7.49
Smoking	−2.82	2.14	0.188	−7.04	1.39

*TEMPS-A*					
Depressive	0.70	0.40	0.078	−0.08	1.48
Cyclothymic	**−0.83**	**0.36**	**0.023**	**−1.54**	**−0.12**
Hyperthymic	0.38	0.25	0.123	−0.10	0.87
Irritable	0.39	0.27	0.796	−0.97	0.74
Anxious	0.39	0.27	0.153	−0.15	0.93

TEMPS-A, Temperament Evaluation of Memphis, Pisa, Paris and San Diego Autoquestionnaire.

## Data Availability

The data underlying this study are available from the corresponding author upon request.
